# Total phenolics, flavonoids, and antioxidant activity of agricultural wastes, and their ability to remove some pesticide residues

**DOI:** 10.1016/j.toxrep.2022.03.038

**Published:** 2022-03-29

**Authors:** Omaima A. Hussain, Emam A. Abdel Rahim, Ahmed N. Badr, Amal S. Hathout, Magdy M. Rashed, Ahmed S.M. Fouzy

**Affiliations:** a Food Toxicology and Contaminants Department, National Research Centre, Dokki, Cairo, Egypt; b Chemical Biochemistry Department, University of Agriculture, Cairo University, Giza, Egypt

**Keywords:** Agricultural waste, Pesticides, Phenolics, Flavonoids, Antioxidant, FTIR, GC/MS/MS

## Abstract

Organophosphorus pesticides (OPPs) cause great risk to human health as they are used globally. Therefore, the purpose of this research was to determine the total phenolics, flavonoids, and antioxidant activity of agricultural waste, as well as to control the pesticide residues (diazinon, and parathion) at a laboratory scale level using dried-milled fruit wastes. The pesticide residues parathion and diazinon were used at concentrations of 0.094, and 1.90 mg/mL respectively. The fruit wastes used in this study were orange and banana peels, as well as date stones, and they were used in two concentrations (3 and 9 g/30 mL deionized water). The total phenolic and flavonoid contents and the antioxidant activity were measured in fruit wastes. Also, the Fourier transmitted infrared (FTIR) spectra of fruit wastes were established to figure out the nature of the functional groups found before and after pesticide residues removal. The ability of fruit wastes to remove pesticides residues was determined using Gas Chromatography/Mass spectrometry (GC/MS). Data showed that date stones contained a higher amount of total phenolic content than orange and banana peels. However, orange peels contained a higher amount of total flavonoid contents than those of date stones and banana peels. As for antioxidant activity, banana peels recorded the higher antioxidant activity, followed by orange peels and date stones respectively. Results revealed that there was no relation between total phenolic content, total flavonoid content, and antioxidant activity. Results also indicated that date stones at a concentration of 9 g successfully reduced diazinon (81.18%), followed by banana (63.86%) and orange peels (43.42%) respectively, whereas parathion was reduced by banana peels at a concentration of 9 g (50.34%), followed by orange peels (45.28%), and date stones (39.52%) respectively. This study demonstrated that agricultural wastes were effective in the adsorption of diazinon from water, and their use is considered safe for the environment.

## Highlights

• Date stones successfully reduced diazinon by 81.18%, whereas banana peels effectively reduced parathion by 50.34%.
• FTIR spectra of fruit wastes displaced severeal peaks.
• Date stones contained a higher amount of total phenolic content.
• Orange peels contained a higher amount of total flavonoid contents.
• Banana peels recorded the higher antioxidant activity.

## Graphical Abstract

**Figure:**
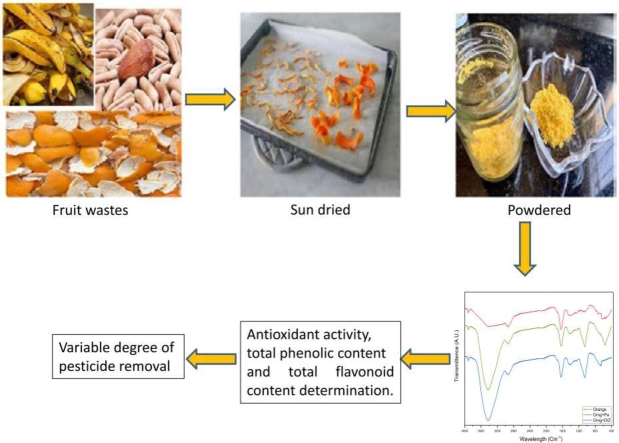

## 1 Introduction

Pesticides are widely used in agriculture to increase crop yield and control pests. [1]. Therefore, pesticides can remain on crops, and in the environment, whereas they have been connected to a variety of environmental contaminations, including soil and water contamination, as well as air pollution [2]. The use of pesticides has had a significant influence on beneficial insects [3]. It was reported that while just 1% of overall pesticides are successful in controlling insect pests on target plants [4], the remaining pesticides breach or attain non-target plants and the environment in significant portions, and this may lead to pesticide pollution which could damage the environment and have a significant consequence on human health [5].

Occupational exposure to pesticides is ubiquitous among farmers worldwide [6] and its adverse effects on human health are a well-documented threat [7], [8]. A variety of acute and chronic poisoning cases around the world has occurred due to pesticide use, such as diabetes, respiratory failures, fertility issues, cancer, and even death [9], [10]. This is considered consistent with that of the European food protection authority which published a document in 2017, indicating that about 44% of regularly produced food contained one or more pesticide residues [11].

In recent years, organochlorine pesticides have been gradually replaced in favor of more efficient, safer chemicals with faster biodegradation rates, such as organophosphorus insecticides [12], [13]. Nevertheless, it has been determined that organophosphorus have a slight endurance (weeks) and may scatter within the environment for a long period [14]. Therefore, there is an urgent need to use natural products to remove pesticide residues from food and drink. Agriculture activities of those remaining from fruit generate a significant amount of waste [15], and the production of a massive quantity of agricultural waste initiates critical environmental problems [16].

Orange peels are largely made up of cellulose, pectin, hemicellulose, lignin, chlorophyll pigments, and other low molecular weight hydrocarbons with multiple hydroxyl functional groups, making it a potential sorbent for a variety of contaminants [17]. Orange peels were found to remove carbofuran from an aqueous solution by adsorption [18]. Similar to orange peels, the primary components of banana peels also included cellulose, hemicellulose, pectin, chlorophyll, and other low molecular weight species [19]. Silva et al. [20] demonstrated that banana peel proved to be a suitable bio-sorbent for atrazine and ametryne removal from waters. The high adsorption capacity of banana peels for metals and organic chemicals is mostly owing to the presence of the pectin's hydroxyl and carboxyl groups [21]. Numerous studies have shown that fruit peels have high phenolic content and antioxidant activity than flesh [22], [23].

Date stones have been shown to have high nutritional value, particularly in terms of fiber and antioxidant content [24]. El-Bakouri et al. [25] stated that more than 90% of aldrin, atrazine, chlorpyrifos, chlorfenvinphos, dieldrin, alpha-endosulfan, endrin, hexachlorobenzene, beta-HCH, gamma-HCH, simazine, and trifluralin were removed by date stones through adsorption.

It could be observed that the removal of diazinon and parathion by orange and banana peels and date stone was not previously studied. Therefore, this study aimed to determine the total phenolics, flavonoids, and antioxidant activity of agricultural waste, as well as to control the pesticide residues (diazinon, and parathion) at a laboratory scale level using dried-milled fruit wastes. This experiment was performed as a simulated model to predict the behavior of these agricultural wastes for the removal of pesticide residues from irrigation waste water. The success of this system would provide a novel strategy to solve the pesticide residues issue, as well as, save the environment against contamination.

## 2 Materials and methods

### 2.1 Chemicals

Diazinon and parathion pesticides were purchased from Santa Cruz Biotechnology Inc., (Santa Cruz, CA 95060, USA).

### 2.2 Fruits

About 10 kg of each of the following fruits were obtained for the experiments. Ripened fresh fruits of sweet orange of Washington Navel orange (*Citrus sinensis* L.), and banana (*Musa* sp) were obtained from the farms of the Egyptian Ministry of Agriculture on December, 30th 2020. Dates (*Phoenix dactylifera* L.) were obtained from a date farm in El-Ayat, Giza on January 4th, 2021.

### 2.3 Preparation of fruit peels

Fruits were checked for defects, insect damage, disease, surface color change, and other defects to ensure the final product’s quality. Fruits were properly washed with distilled water (LWDB-400 M, Laboid International, India) to remove any dust or debris that had adhered to the peels and then wiped dry. Fruit peels were separated manually and cut into small parts for about 2 × 2 cm, then sun-dried for 96 h. The fruit peels were then ground thoroughly by a house mill and passed through a 0.25 mm mesh.

### 2.4 Preparation of Date stones

Date stones were cleansed with water to remove any adhered date flesh before being sun-dried. A heavy-duty mill (M20 Universal Mill, IKA®-Werke GmbH & Co. KG, Germany) was used to grind the collected date stones. The powder was then passed through a 0.25 mm mesh.

### 2.5 Removal of pesticide residues

Diazinon and parathion were added separately at a concentration of 1.90 and 0.094 mg/mL respectively to water containing each of the following separately; 1) ground/milled orange peels 2) ground/milled banana peels, and 3) ground/milled date stones at concentrations of 3.0 and 9.0 g/30 mL deionized water. Larger agricultural waste quantities were not evaluated to avoid an overabundance of biosorbent, which would limit practical uses. Positive controls were prepared to contain each of the pesticides separately without the ground/milled agricultural wastes. Negative controls were prepared to contain each of the ground/milled agricultural wastes separately, without pesticides. The samples were shaken in a shaking incubator (ZWYR-211D, Shanghai ZHICHENG Analytical Instruments Manufacturing Co., China) (150 rpm, 20 min, 27 °C). The suspensions were centrifuged (Labnet International Inc., USA) at 1750 × *g* to remove the agricultural waste. The samples were filtered, and the filtrated solutions were analyzed for the pesticide residues using GC/MS/MS [20].

### 2.6 Gas Chromatography-Tandem Mass Spectrometry (GC/MS/MS) analysis

Gas Chromatography system 7890B with tandem mass spectrometer 7010A Quadrupole (Agilent Technologies, USA) was used. Chromatographic separations were achieved using the HP5MS ultra inert capillary column (30 mm × 0.25 mm, 0.25 µm). Mass Hunter software version 7.01 (Agilent Technologies, USA) was used for instrument control and data acquisition/processing. Under scanning settings, a mass spectral library (NIST 14) was utilized to confirm pesticides and identify co-extractives. The GC oven temperature was programmed to initially be held at 70 °C for 1 min then ramped to 150 °C at 50 °C/min for 0 min, and raised to 260 °C at the rate of 6 °C/min for 0 min, then ramped from 260 to 310 °C at 20 °C/min for 1.567 min with a total run time of 25 min. In splitless mode, one microliter of each sample was injected, and detection was achieved using electron impact ionization in positive ion mode (70 eV). At a flow rate of 1.654 mL/min ultra-high purity helium (> 99.999%) functioned as the carrier gas. The flow rates of collision cell gases (helium, quench gas; nitrogen, collision gas) were 2.25 mL/min and 1.5 mL/min, respectively. The temperatures of the injector, transfer line, ion source, and quadrupole were 250, 280, 300, and 180 °C, respectively. During solvent delay time of 2 min, the filament current (100 μA) was switched off. The acquisition method was utilized in MRM mode, with one MRM transition used for quantification (quantifier peak) and the others for confirmation (qualifier peaks). Diazinon and parathion standards were used.

### 2.7 Determination of active group using Fourier-transform infrared spectroscopy (FTIR)

Absorption spectroscopy in the infrared region (400–4000 cm^-1^) at 4 cm^-1^ resolution was used to analyze the functional groups contained in dried orange and banana peel, as well as date stones [26]. The FTIR spectra were captured using a spectrometer (Bruker, USA). To reduce spectrum contributions from ambient carbon dioxide and water vapor, the FTIR spectrometer was purged. The mean of four spectra from separate pellets of the same sample was then computed.

### 2.8 Preparation of fruit waste extract

The milled peels of oranges and banana, as well the milled date stones were extracted using Ultra-sonic (Smith Lab., Jainsons India Regd., India) assist in line with the methodology explained by Vinatoru et al. [27]. One hundred g of dried powder was weighed, and then solvent extraction with aqueous methanol (80%) was carried out. Extraction was carried out using an ultrasonic water bath (30 min; 5 W/cm^-2^) (Smith Lab., Jainsons India Regd., India). The temperature was kept constant (25 °C). A rotary evaporator (LabTech S.r.l., Italy) was then used to evaporate the extracts at room temperature. The recovered residues were re-evaporated to eliminate contaminants and kept at 4 °C until analysis.

### 2.9 Determination of total phenolic content

Folin-Ciocalteu’s reagent was used for the determination of total phenolic content, with gallic acid as a standard. At 765 nm, the absorbance was measured, and results were articulated as (mg gallic acid equivalent (GAE) per g) [28], [29].

### 2.10 Determination of total flavonoid content

The aluminum chloride colorimetric method was used for the determination of total flavonoid contents with quercetin as standard. At 533 nm, the absorbance was measured, and the results were presented as (mg quercetin equivalents (QE) per g) [30].

### 2.11 Determination of antioxidant activity

#### 2.11.1 Antioxidant activity using 2, 2-diphenyl-1-picrylhydrazyl (DPPH) assay

The DPPH was measured according to the method designated by Abdel-Razek et al. [31], and Badr et al. [32]. An IC_50_ was also calculated, which indicated the quantity of fruit wastes (mg) in 1 mL of solution needed to reduce the initial concentration of DPPH radicals by 50%, with ascorbic acid as a standard. The results were articulated as (mg of ascorbic acid equivalent antioxidant activity (AEAC) per 100 g).

#### 2.11.2 Antioxidant activity using 2, 2′-azino-bis (3-ethylbenzothiazoline-6-sulfonate) (ABTS) radical scavenging

ABTS radical scavenging experiment was carried out according to Fitriana et al. [33]. At a wavelength of 734 nm, the absorbance of the samples was measured, with ascorbic acid as a standard. The results were expressed as (mg ascorbic acid per 100 g).

#### 2.11.3 Reducing power antioxidant activity (RPAA)

A modified spectrophotometric approach according to the method of Ferreira et al. [34] was applied. At 700 nm, the absorbance was measured spectrophotometrically, with ascorbic acid serving as a standard [35], [36]. The results were represented as (mg ascorbic acid equivalent (AA) per 100 g).

### 2.12 Statistical analysis

The data were presented as mean (95% CI). Statistical analysis was performed using the SPSS software version 16. A one-way analysis of variance (ANOVA) was carried out, in which *P* < 0.05 was regarded statistically significant. All tests were performed in triplicate (*n* = 3).

## 3 Results and discussions

### 3.1 Removal of pesticide residues

Results in Table (1) showed the impact of fruit waste on the reduction of pesticide residues in water. Results revealed that date stones at a concentration of 9 g successfully reduced diazinon (81.18%), followed by banana (63.86%) and orange peels (43.42%) respectively. On using fruit waste at a lower concentration (3 g), banana peels showed a higher ability to reduce diazinon, followed by date stones and orange peels respectively. On the other hand, the reduction of parathion was accomplished using fruit waste at a concentration of 9 g in the following order; banana peels (50.34%), orange peels (45.28%), and date stone (39.52%) respectively. At a lower concentration (3 g), parathion was highly reduced by banana peels, followed by orange peels and date stones respectively. In agreement, Silva et al. [20] demonstrated that banana peel is a suitable bio-sorbent for atrazine and ametryne from waters. Orange peels on the other hand were utilized as a low-cost adsorbent to remove carbofuran [18] and furadan from aqueous solution by adsorption [37]. Pathak et al. [38] stated that a wide variety of fruit peel wastes was exploited for the elimination of various organic and inorganic compounds (dyes, heavy materials, pesticides, etc.). For date pits, Hassan et al. [39] reported that roasted date pits removed profenofos at a concentration of 28 ppm by 11.20%.

**Table 1:** Effect of fruit waste on the percentage pesticide residues reduction.

Fruit waste	Weight (g)	Percentage of reduction (%)	
		Diazinon	Parathion
Orange peels	3	4.71 (3.194–6.226)	20.31 (18.341–22.279)
	9	43.42 (40.964–45.876)	45.28 (42.711–47.849)
Banana peels	3	46.27 (43.950–48.590)	28.73 (27.361–30.099)
	9	63.86 (60.986–66.734)	50.34 (47.613–53.067)
Date stones	3	20.45 (19.047–21.853)	12.51 (11.299–13.721)
	9	81.18 (78.023–84.337)	39.52 (37.755–41.285)

Results are expressed as mean (95% CI).

95% CI: 95% Conﬁdence interval of the mean.

Results showed no significant difference *P* > 0.05.

### 3.2 Fourier transmitted–infrared spectroscopy of fruit wastes

**Fig. 1:**
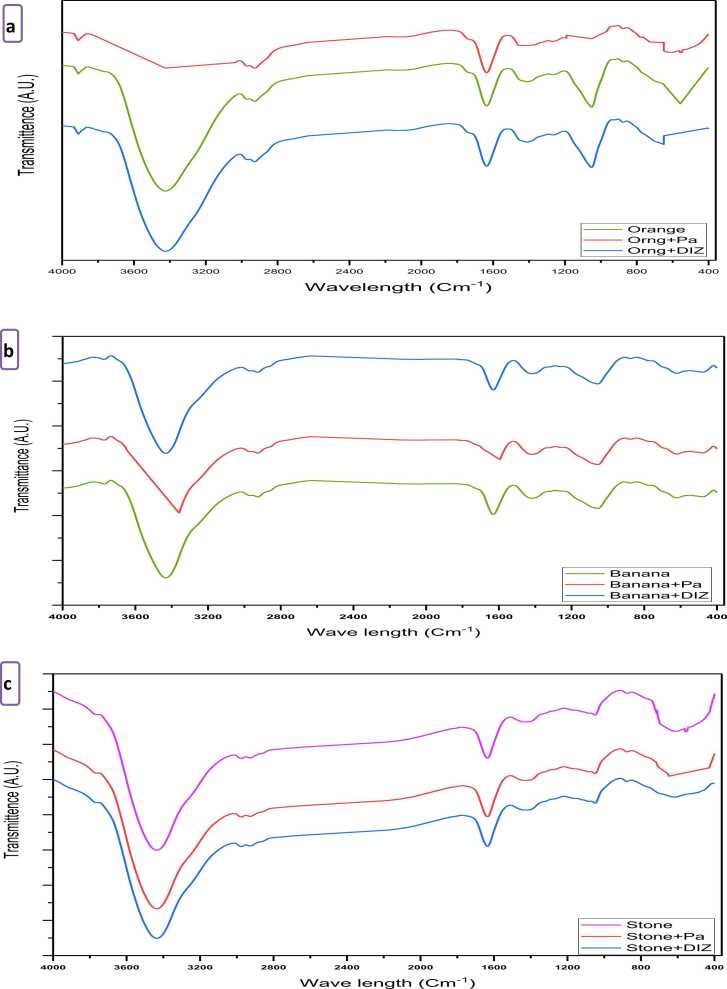
FTIR spectra of (a) orange peels, (b) banana peels, and (c) date stones.

The FTIR spectra of orange and banana peels, as well as date stones, were determined to understand the nature of the functional groups existing on the exterior of the date stones and peels (Fig. 1, Table 2). Data in Fig. 1(a), displayed the peaks that showed the complicated nature of the orange peels, with or without the pesticide residues’ existence. Bands appearing at 3424.96, 2928.38, 1636.3, 1411.64, 1051.98, and 612.288 cm^−1^ were assigned to O-H stretching, carboxylic acids (–OH), C=O stretching of amide, alkane (–CH_3_), C-O stretch, and C-H bending of alkynes, respectively. Similar findings have been stated by El-Nemr et al. [40], and Afolabi et al. [41].

**Table 2:** FTIR of fruit wastes.

IR frequencies (cm^−1^)			Functional groups
Orange peels	Banana peels	Date stones	
3424.96	3432.67	3435.56	Alcohol (OH stretch)
2928.38	2926.45	–	Carboxylic acids (–OH)
1636.30	1630.50	1636.30	Amides (C=O stretch)
1411.64	1421.28	1429.96	Alkane (–CH_3_)
1051.98	1054.87	1050.50	Alcohol (C-O stretch)
612.288	620.002	616.15	Alkynes (C-H bend)

Results in Fig. 1(b) displayed several peaks for the banana peels. Bands appeared at 3432.67, 2926.45, 1630.5, 1421.28, 1054.87, and 620.002 cm^−1^ and were assigned to O-H stretching, carboxylic acids (–OH), C=O stretching of amide, alkane (–CH3), C-O stretch, and C-H bending of alkynes, respectively. In agreement, Memon et al. [42] reported the FTIR spectra of the banana peels displayed several peaks occurring at 3313.4, 2920.3, 2850.6, 1734, 1613.6, 1317.4, 1035.2, and 884.6 cm^−1^. Similar results were reported by Afolabi et al. [43]. Results in Fig. 1(c) displayed the number of peaks for date stones and showed similar functional groups to those of orange and banana peels except for carboxylic acid which was not present in date stones. Similar observations were reported by Hammani et al. [44].

The presence of long-chain fatty acids, waxes, carotenoids, and phytosterols is thought to be consistent with the methylene v(C-H) asymmetric stretch between 2800 and 3000 cm^-1^ [45]. Pectin, cellulose, or lignins are the primary sources of carboxylic acid in fruit peels [46]. Similarly, the vibration at 1630 cm^-1^ suggests the existence of the carbonyls of phytosterols and fatty acids [45]. Glycosidic units were obvious in the range of 950–617 cm^−1^ [47]. Fruit peel organic components mostly consist of cellulose, hemicellulose, pectin molecules, chlorophyll pigments, and other low molecular weight compounds [48]. Most of the bands were common to those observed in cellulose, hemicellulose, and lignin [49].

It is of importance to mention that the presence of pesticide residues led to a change in the appearance of the FTIR spectra, particularly in the part related to the (O-H) groups. The depth of the FTIR spectra of orange peels wavelength of 3424.96 cm^−1^ was decreased in the presence of diazinon, which means consumption of (O-H) groups by reaction or binding. The changes in the FTIR spectra of banana peels due to parathion existence were verified in the wavelength of 1630.5 cm^−1^. The change in the spectra could point out the possible mechanism of fruit waste impact to reduce the pesticide residues. Regarding the dates stones spectra, the changes appeared clearer regarding the presence of diazinon and parathion in date stones. The change of fruit waste spectra means a change in their active functional groups. These slight but significant changes in the FTIR spectrums following pesticide adsorption demonstrated that chemical adsorption of pesticides on fruit waste is achievable [50]. The FTIR graphs of the agricultural wastes revealed a high content of the hydroxyl groups, which may play a significant role in pesticide reduction. Thus, it could be suggested that a chemical reaction occurred between the bioactive constituents of the agricultural wastes and pesticide residues which is supported by the FTIR changes.

### 3.3 Total phenolic content

**Fig. 2:**
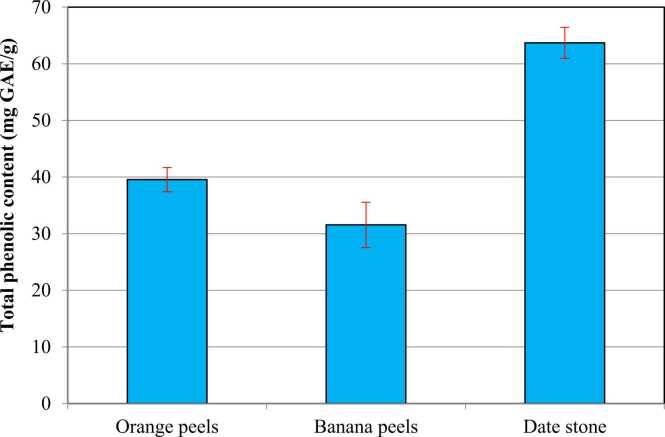
**Total phenolic content of orange peels, banana peels, and date stone.** Results are expressed as mean (95% CI). 95% CI: 95% Conﬁdence interval of the mean. Results showed no significant differences *P* > 0.05.

The total phenolic contents of the orange and banana peels and date stones were expressed as mg of gallic acid equivalent per g dry weight (Fig. 2). Date stones contained the highest concentration of total phenolic content, followed by orange and banana peels. In agreement, Afifi et al. [51] indicated that maximum total phenolic content (71.6 mg GAE/100 g) was determined in date seed. Several studies reported that date seeds displayed higher total phenolic content compared to other fresh and dried fruits [52], [53], [54], [55]. Concerning the total phenolic content in orange peel, M’hiri et al. [56] noticed that orange peel dried at 100 °C had the highest total phenolic content (65.72 ± 3.42 GAE mg/g) compared to the fresh peel (39.45 ± 1.00 GAE mg/g). These results were considered higher than our study. Recently, Pandey et al. [57] revealed that the total phenolic contents for citrus peels varied from 6.0 to 16.09 mg GAE/g, which is considered lower than our study. Total phenolic content in banana peels was considered higher than those described by Aboul-Enein et al. [58] who reported that total phenolic content in banana peels recorded 17.89 mg GAE/g.

### 3.4 Total flavonoid content

**Fig. 3:**
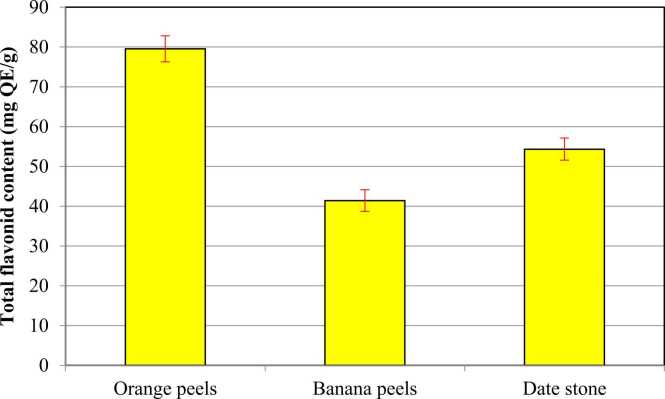
**Total flavonoid content of orange peels, banana peels, and date stone.** Results are expressed as mean (95% CI). 95% CI: 95% Conﬁdence interval of the mean. Results showed no significant differences *P* > 0.05.

Data in Fig. (3) described the total flavonoid content in the orange and banana peels and date stones, and they were expressed as mg quercetin equivalents per gram. The obtained results indicated that orange peels contained a higher amount of the total flavonoids than those of date stone and banana peels. Similar findings were stated by Sir Elkhatim et al. [59] who revealed that the total flavonoids in orange fruit peels were 83.3 mg/g. Flavonoids, the most common components in citrus peels, have been shown to exhibit a wide range of antioxidant properties [60]. For date stones, Herchi et al. [61] reported that total flavonoid levels were observed to vary in date seeds. In agreement, Bouhlali et al. [62] stated that date seeds exhibited high flavonoid contents. Banana peels showed lower total flavonoid content. In agreement, Kurhade et al. [63] reported that ethanolic banana peel extract contained total flavonoid content at a concentration of 29.5 mg/g.

### 3.5 Antioxidant activity

**Fig. 4:**
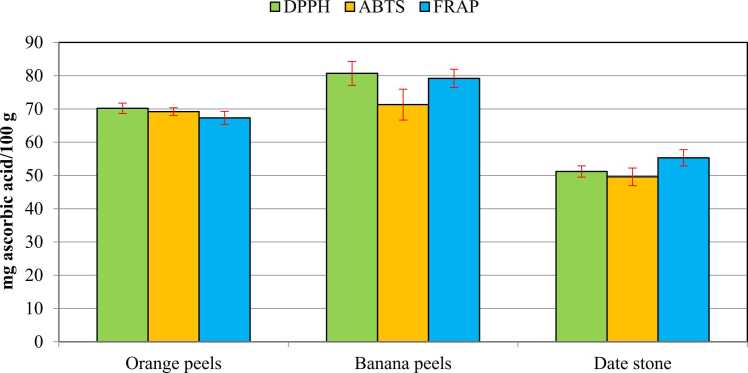
**Antioxidant activity of orange peels, banana peels, and date stone.** Results are expressed as mean (95% CI). 95% CI: 95% Conﬁdence interval of the mean. Within different fruits, results showed significant differences *P* < 0.05. Within different antioxidant studies, results showed no significant differences *P* > 0.05.

**Fig. 5:**
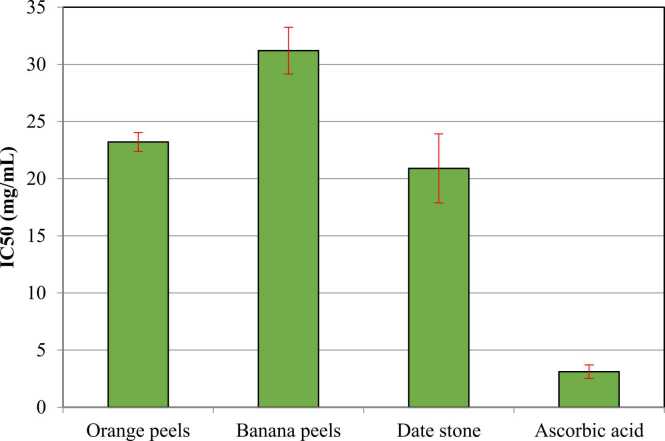
**Antioxidant activity showing the IC**_**50**_**of orange peels, banana peels, and date stone.** Results are expressed as mean (95% CI). 95% CI: 95% Conﬁdence interval of the mean. Results showed significant differences *P* < 0.05.

Antioxidant activity of the fruit peels and date stones was determined using three different assays (Fig. 4), as well as the IC_50_ value, which was also estimated against ascorbic acid as a standard reference (Fig. 5). Results in Fig. (4) revealed that banana peels recorded the higher antioxidant activity, followed by orange peels and date stones respectively. Similar results were reported for the IC_50_ values. The presence of several antioxidant components may be responsible for the remarkable antioxidant capabilities of banana peels [64]. Aboul-Enein et al. [58] found that the reducing power of banana peel is most likely due to the action of the phenolic compounds' hydroxyl groups, which may act as electron donors. On the other hand, citrus peels are a rich source of naturally occurring antioxidants, which have promising antioxidant activity [65]. On studying the antioxidant activities of a Moroccan date stone, antioxidant activities were estimated using three assays (FRAP, DPPH, and ABTS radical scavenging activities) [66]. The antioxidant activity of the evaluated agricultural wastes showed variations, whereas the highest antioxidant activity was for banana peels. Although the total flavonoid and total phenolic content of banana peels were fairly low, their antioxidant activity was higher, implying that anti-oxidative substances other than flavonoids and phenolic have participated [67]. The antioxidant activity of the evaluated agricultural wastes showed variation with the high potency of banana peels.

**Fig. 6:**
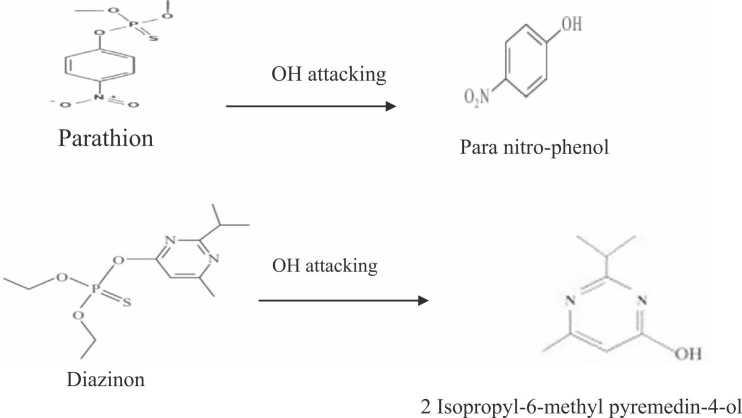
The suggested reduction mechanism of parathion and diazinon according to agricultural waste constituents’ activity.

Several studies have found that pesticide exposure can cause oxidative stress by increasing the formation of free radicals, which can accumulate in the cell and damage biological macromolecules [68], [69]. The agricultural wastes showed richness in their phenolics (date stones), and flavonoids (orange peels). These polyphenols and flavonoids might have acted as antioxidants to protect against free radicals produced by the pesticide residue. Another mechanism suggested for the removal of pesticide residues is the ability of the hydroxyl group to attach to some molecules containing oxygen and bind together [70]. This process has led to the transformation of parathion and diazinon into less harmful compounds such as para-nitro phenol and 2 isopropyl-6-methyl pyrimidine-4-ol respectively ( Fig. 6). Thus, it is necessary to add these natural products to the soil to provide safe crop production.

There has been little research on the use of agricultural wastes for the removal of pesticides residues from water. Water quality has been a major source of concern in recent years, as it is one of the most important needs of humans, and animals [71], and the availability of safe and high-quality water has long been a goal of human culture [72]. Therefore, the current study represents a novel technique for the removal of pesticide residue from the water. Pesticides are classified as highly dangerous chemical substances, the consequences of which are felt by humans, animals, and the ecosystem as a whole. The study discusses how to get rid of those pesticide residues by using agricultural wastes. Agricultural wastes have an economic dimension as they are of low cost, and an environmental dimension because the accumulation of these agricultural wastes causes environmental problems. The use of agricultural waste as a natural product, extracts, or even as a substance with a chelating character to environmentally harmful substances is of great importance. Thus, the implementation of agricultural wastes will have scientific significance.

The research's strength was studying the removal of pesticide residues by agricultural wastes, which received little attention, in contrast to the use of activated carbon prepared from agricultural wastes which received more attention [20], [73], [74]. The limitation of this study is the use of a large amount of agricultural wastes (more than 9 g), which may limit their practical application, for example in soil, leading to an overabundance of bio-sorbent, and also changes in the soil characteristics.

Future research should include the detection of suitable and lower quantities of agricultural waste, as well as other agricultural wastes than those studied here. Future research should include the application of these agricultural wastes in the soil to study the removal of pesticides residues in soil.

## 4 Conclusion

Date stones extract recorded higher total phenolic contents; whereas orange peels extract recorded higher total flavonoid contents. On the other hand, banana peels extract recorded higher antioxidant activity. These results indicated that the enrichment of these agricultural wastes with phytochemicals might have provided a modern approach for reducing pesticide residues. The FTIR of agricultural wastes reflected significant changes after their exposure to the pesticide residues; this might be due to the removal of pesticide residues. More studies are required for the detection of suitable and lower quantities for the application of each fruit waste for the reduction of several pesticide residues. In this regard, the results of this study recommend date stones to be used to solve the commonness of pesticide residues in water.

## CRediT authorship contribution statement

**Omaima Hussain:** Conceptualization, Methodology. **Emam Abdel Rahim:** Supervision. **Ahmed Badr:** Data curation, Visualization. **Amal Hathout:** Writing – review & editing. **Magdy Rashed:** Supervision. **Ahmed Fouzy:** Project administration, Funding acquisition.

## Declaration of Competing Interest

The authors declare that they have no known competing financial interests or personal relationships that could have appeared to influence the work reported in this paper.
